# One Environmental Health: an emerging perspective in toxicology

**DOI:** 10.12688/f1000research.14233.1

**Published:** 2018-06-27

**Authors:** Adam Pérez, John Pierce Wise Sr.

**Affiliations:** 1 Wise Laboratory of Environmental and Genetic Toxicology, Department of Pharmacology and Toxicology, School of Medicine, University of Louisville, 505 S. Hancock Street, Louisville, KY 40292, USA

**Keywords:** One Health, chromium, endocrine disruptors, carcinogenesis, metals, whale, alligator, DNA repair

## Abstract

The One Environmental Health research approach, a subspecialty of the One Health initiative, focuses on toxic chemicals. Distinct disciplines work together to give a holistic perspective of a health concern through discrete disciplines, including, but not limited to, public health and the medical and veterinary sciences. In this article, we illustrate the concept of One Environmental Health with two case studies. One case study focuses on alligators and contributions to the field of endocrine disruption. The other case study focuses on whales and contributions to understanding carcinogenic metals. Both studies illustrate how the health of sentinel organisms has the potential to inform about the health of humans and the ecosystem.

## Introduction

**Figure 1.**
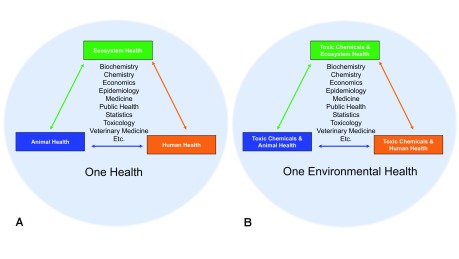
One Environmental Health interconnects human health, wildlife health, and ecosystem health as one. ( **A**) Schematic showing the interconnectedness of the larger concept of One Health. ( **B**) Schematic showing the interconnectedness of the specific concept of One Environmental Health, the subset of One Health that focuses on toxic chemicals, as showcased in this review by case studies on alligators and whales. The data from humans, alligators, and whales help inform on potential common hazards and even provide insights into the mechanism of action of those hazards. Conversely, the data from the latter subjects can be extrapolated to understand potential risks to organisms that make up an ecosystem. A loss of fitness of even one organism within an ecosystem will affect both biotic and abiotic components. One Environmental Health’s holistic approach can provide better research strategies to tackling present and future hazards to everyone’s health.

Current research practice typically involves a singular focus on human health, on animal health, or on ecosystem health. This approach implies there are three separate and distinct “healths”, one for each group, and it misses important potential discoveries that can be gleaned by investigating the interconnectedness of health. In contrast, One Health posits there is actually only “one” health shared by humans, animals, and the ecosystem and what affects one affects all three ^1, 2^ ( Figure 1A).

One Health is a growing and important area of research, yet many are unfamiliar with its concepts. Simply put, One Health focuses on the interconnectedness of human, animal, and wildlife health in order to provide a deeper understanding of health. At first glance, this concept of interconnectedness seems like common sense, and, as a general concept, it has a long history with roots in ancient Egypt, ancient China, and ancient Greece ^3^. However, as a research approach and a practical matter, One Health is just beginning to be understood and emphasized.

One Health brings together a broad spectrum of expertise, including medical, veterinary, biological, epidemiological, and toxicological sciences, among others. One Environmental Health is the subset of One Health that focuses specifically on toxic chemicals ( Figure 1B). It includes aspects related to climate change and other non-chemical concerns but narrows their focus in this subspecialty to how they affect the availability and impact of toxic chemicals. The purpose of this review is to illustrate the concept and approach to One Environmental Health with two different, contemporary case studies. For clarity and simplicity, we chose case studies that each included all three aspects of the One Health triad (human health, animal health, and ecosystem health) studied in a singular type of species (alligators and whales). We use these case studies as a tool to show the One Environmental Health concept in practice; however, there are multiple approaches to One Environmental Health, which could include multiple species, multiple toxic endpoints, or findings from multiple laboratories, each working on a different species, among others. In this review article, we present two case studies: one focused on alligators and reproductive toxicology and one focused on whales and mechanistic carcinogenesis.

## Alligators and endocrine disruption

One example of the One Environmental Health approach is the case of endocrine-disrupting chemicals (EDCs) and reproductive health challenges highlighted by research on alligators. Lake Apopka is located in the central region of Florida and is the third largest lake in the state ^4^. Prior to the 1940s, the lake maintained a quasi-pristine state free of most environmental pollutants, despite a canal dug in the 1890s which lowered its elevation and thus altered the surface water outflow of the lake ^5^. Anthropogenic effects in the late 1940s severely compromised the diverse ecology the lake had maintained, after much of the marshland north of the lake (approximately 12,960 hectares) was repurposed for agriculture. Additionally, a new inflow of effluent discharges arose from the city of Winter Garden’s sewage treatment plant and a citrus processing plant ^5^. These developments led to Lake Apopka becoming one of Florida’s most polluted lakes ^6^. It did not take long to notice the effects of runoff chemicals on one of Florida’s most iconic residents: the American alligator ( *Alligator mississippiensis*). While the population of the American alligator was thriving elsewhere in Florida, after the Endangered Species Act of 1973 protected it, the alligator population at Lake Apopka was experiencing apparent decreases in fertility and increases in embryonic deformities ^6– 8^. Studying these animals provided important lessons about alligator health, human health, and ecosystem health.

### Alligators inform about alligator health

The initial insights into what was affecting the fertility of alligators in Lake Apopka came from alligator eggs ^4– 9^. Eggs were collected from Lake Apopka and Lake Woodruff (used as a control, quasi-pristine lake). The eggs were artificially incubated to determine viability, and the neonates were reared for collection of blood and tissue samples. Eggs from Lake Apopka on average showed decreased viability and neonates also presented higher mortality rates than those observed in the specimens from Lake Woodruff ^4^. Two blood sample collections were taken from robust 6-month-old alligator juveniles whose eggs originated from Lake Apopka and Lake Woodruff. Plasma was tested for estradiol-17β and testosterone. Juveniles were culled for morphological and histological examination of the gonads. It was found that female alligators from Lake Apopka had higher plasma estradiol-17β concentrations when compared with females from Lake Woodruff or males from either lake ^4^. Furthermore, males from Lake Apopka presented a mean plasma testosterone concentration fourfold lower than their counterparts from Lake Woodruff. In fact, the mean plasma testosterone concentration for the male alligators from Lake Apopka was comparable to the levels found in females from both lakes ^4^.

Gonadal development was erratic in some of the specimens from Lake Apopka ^4^. Some males were misidentified as females because of a severe decrease in phallus size. In addition, some females had clitoral enlargement comparable to the size of a properly developed male phallus. Testes and ovaries initially presented proper morphological attributes for animals at the surveyed development age, but upon cytological inspection, there were some obvious differences. For example, ovaries from Lake Apopka individuals presented a large number of polyovular follicles and polynuclear oocytes, something not present in any of the specimens from Lake Woodruff. In males, the testes from individuals originating from Lake Apopka had many anomalous cell morphologies within the seminiferous tubes ^4^. Studies corroborated organochlorine compounds found in Lake Apopka did in fact competitively inhibit the binding of estradiol-17β to the estrogen receptor from protein isolates derived from female alligators ^10^. The latter studies ^10– 12^ complemented the correlation between these chemicals and the reproductive fitness of American alligators with potentially sound evidence of a definitive cause-and-effect relationship.

A study by Kohno *et al.* ^13^ show altered mRNA expression of genes related to gonadal development (nuclear receptor subfamily 5 group A member 1, or **** *NR5A1*), steroid hormone synthesis (steroidogenic acute regulatory protein, or *STAR*), estrogen receptors, folliculogenesis, and ovarian development (aromatase and *DAX1*) in alligators from Lake Apopka. Furthermore, Helbing *et al*. ^14^ showed a correlation between steroid and thyroid hormones, and the expression of their respective receptors, to ovary function and development. Overall, these alligator studies collectively made it clear that pesticides were affecting the health of the alligators.

### Alligators inform about human health

Sentinel organisms respond to disturbances of varying nature (chemical, biological, physical, and so on) at levels low enough to avoid a negative outcome in humans and provide an early warning mechanism for such hazards ^15^. Reptiles are good sentinels of exposure to endocrine-disrupting contaminants owing to their lability in sex determination. Minute amounts of steroids, steroid-mimicking compounds, or steroid synthesis-disrupting compounds can induce permanent effects even during low-exposure scenarios as long as exposure occurs in critical stages of sex determination ^11^. Alligators, being on the top of the food chain in their niche and having relatively long life spans ^16^, are suitable sentinel organisms for understanding how a contaminant traverses the lower trophic levels and ultimately affects the ecosystem as a whole ^17^. This new insight into the effects of environmental steroid-mimicking chemicals, such as ecoestrogens ^18^, has also brought attention to the need to translate findings in organisms such as the American alligator to humans ^19^. Alligator studies are a clear example of how wildlife studies connect with the field of human health, as similar reproductive effects seen in wildlife subjects were also observed in human populations ^19^. For example, a population-wide study in Paris, France showed an incidental decline in semen quality and genital abnormalities, which did not correlate with specific demographics and which could only be attributed to an environmental contaminant in the area that affected sex development ^20^. Consistent with the hypospadia malformation of the penis observed in American alligators in Lake Apopka ^4^, humans also presented reduced penile lengths correlated with *in utero* exposure of diethylstilbestrol (DES) ^21^. Estrogen and progesterone receptors found in the oviduct of female American alligators were studied in a competition-binding assay, in parallel with human homologs, using a battery of environmental chemicals (for example, toxaphene and chlordane). The chemicals showed similar synergistic effects for inhibiting natural estradiol-17β binding in receptors from both alligators and humans ^22^.

Endocrine disruptor studies expanded to include studies beyond estrogenic, androgenic, anti-androgenic, and anti-thyroid actions ^22– 24^. Studies include expression and regulation beyond proteins. Katsu *et al.* studied direct transcriptional regulation of estrogen and progesterone receptors after intravenous injection of exogenous estradiol-17β ^25^. Sexually dimorphic gene expression was also affected in hatchlings from Lake Apopka (undistinguishable between male and female subjects) when compared with non-contaminated eggs from control lakes, which were reared for one year in a controlled laboratory setting ^26^. It was further postulated that endocrine disruptors (for example, organochlorines) can influence gene expression and regulation through epigenetic means in human reproductive organs, particularly the ovaries ^27^. Persistent organic pollutants, such as the dichlorodiphenyldichloroethylene (DDE) in Lake Apopka, have been correlated with global DNA methylation changes in blood samples of Greenlandic Inuit exposed to high levels of these chemicals ^28^. In another example, a study by Kim *et al*. ^29^ showed that a subset of a healthy Korean population exposed to low levels of organochlorines exhibited decreased global DNA methylation from blood samples. The role of epigenetics indicates a potential transgenerational effect for endocrine disruptors ^30– 32^ that suggests offspring may be affected for generations even after sites like Lake Apopka become contaminant-free.

Studies from the American alligators in Lake Apopka may also shed light on human reproductive illnesses, such as polycystic ovary syndrome, in which affected females experience altered ovarian gene expression, abnormal ovulation, and the incidence of metabolic diseases ^33^. Moore *et al.* ^34, 35^ discovered female alligators from Lake Apopka presented lower mRNA expression of important ovarian regulatory genes aromatase (converts androgens to estrogen) and follistatin (important for folliculogenesis) even after stimulation by follicle-stimulating hormone (FSH). Similar parallels to exposure to EDCs could explain similar human diseases.

### Alligators inform about ecosystem health

All wildlife is intrinsically interconnected within and beyond their natural niches. The loss of fitness of one component will inevitably have far-reaching consequences in the overall ecosystem that might not be able to recover rapidly. EDCs hinder the success of the ecosystem fauna by affecting reproduction, especially in long-lived organisms such as alligators and humans. Alligators are an example of an organism that does not have functional redundancy ^36^ in its habitat (that is, the role of alligators on the top of the food chain could not be occupied by another organism in Lake Apopka), and this makes this organism even more crucial to the ecosystem functional equilibrium. Almost two decades of studies with American alligators have produced important information about ecosystem health of wetlands in the southern United States (US) ^37^.

The pioneering work on endocrine disruptors performed on American alligators translates to other fauna, such as the Mosquitofish ( *Gambusia holbrooki*) in Lake Apopka, which have also shown male phallus dysmorphism and reduced sperm count ^38^, that are also exposed to similar xenobiotics. Bird and mammal endocrine receptors exhibit overall protein sequence homology and hormone and DNA-binding domain homology with alligator receptors ^25, 37^. Furthermore, vertebrates have largely conserved reproductive systems and molecular mechanisms ^39^. This means many of the effects by EDCs observed on the sentinel American alligator have the potential to affect other wildlife in a similar fashion, especially after chronic exposure to a mixture of EDCs.

## Great whales, metal pollution, and mechanistic chemical carcinogenesis

A second example of the One Environmental Health approach is the case of metal pollution and mechanistic chemical carcinogenesis highlighted by research on great whales. Whales are cosmopolitan animals with some species occupying the ocean all over the world. They also include the largest animal on earth (blue whale [ *Balaenoptera musculus*]) and the longest-lived mammal (bowhead whale [ *Balaena mysticetus*]).

Whales are long-lived marine mammals ^40^ with low reproductive rates ^41, 42^. Many whale species are endangered or threatened. Some are severely endangered, such as the North Atlantic right whale ( *Eubalaena glacialis*), which has a population of about 400 individuals ^43^, and the extremely endangered North Pacific right whale ( *Eubalaena japonica*), which has a population of roughly 25 individuals in the Bering Sea ^44^. Losses to whale populations from boat strikes and fishing gear entanglements are well-understood threats ^45, 46^. However, these cannot explain the low reproduction rates some species have, which are more likely exacerbated by other stressors such as chemical and noise pollution.

The great whales also appear to have very low cancer rates. For example, one study ^47^ found only two neoplasms (both benign) in 2,000 whales (a rate of 0.1%) whereas another study ^48^ found only 14 tumors in 1,800 cetaceans (a rate of 0.08%). Indeed, a recent review of marine mammal neoplasia found only 31 documented cases ever reported in great whales, and most were benign tumors ^49^. These observations and their worldwide distribution make whales interesting subjects to study the impact of carcinogenic metals.

### Whales inform about whale health

Data show increasing levels of pollution in tissues from marine mammals. Metals, in particular, are a growing concern, and data show whales are exposed to high levels of metals, such as chromium (Cr), as shown by skin tissue concentrations obtained from biopsies of healthy free-ranging whales ^50– 54^. Metal toxicology in whales is generally poorly understood, largely because of a lack of controlled toxicology studies in whale-specific models and difficulty in accessing the animals themselves. Cell cultures are a common tool to assess health threats. For example, genotoxicity studies in cell cultures are commonly used by government agencies to help assess health risk ^55^. Metal toxicology, specifically focused on Cr, has been studied in whale cell culture in recent years.

Cr is a known human carcinogen that causes DNA damage and affects reproduction and development ^56– 58^. Cell culture studies from whale biopsies demonstrate that Cr accumulates in cells and affects cell fitness. Cytotoxicity assays in fin whale ( *Balaenoptera physalus*) skin fibroblasts show a decrease in relative survival correlates with an intracellular concentration-dependent increase of Cr from both soluble and particulate sources ^59^. In the same study by Wise *et al.*, increased intracellular Cr resulted in a concentration-dependent increase of chromosomal aberrations. A similar study by Wise *et al.* ^60^ performed on sperm whale ( *Physeter macrocephalus*) skin fibroblasts, a toothed whale, show similar concentration-dependent cytotoxic and genotoxic effects when exposed to Cr. The concentration of Cr used in both studies simulates biologically relevant Cr concentrations based on the total Cr levels calculated from environmental whale biopsies.

Cr can accumulate in testes ^61^, and a study by Wise *et al*. ** ^59^ shows hexavalent chromium [Cr(VI)] exposure is cytotoxic and genotoxic to whale testis fibroblasts. In addition, fibroblasts from whale testes were more susceptible to cytotoxicity than were whale lung fibroblasts at the same Cr(VI) concentration ^59^. Previously, human studies showed chronic Cr(VI) exposure correlates with decreased sperm fitness and motility ^62, 63^, although no definitive mechanism has been elucidated. Thus, taken together, these data suggest that Cr(VI) exposure may cause reproductive dysfunction in whales and is potentially a factor in the low reproductive rates.

### Whales inform about human health

Studies in whale cells may lead to provocative new insights into genotoxic and carcinogenic mechanisms and how to prevent them. Cr(VI) is a well-established human carcinogen, but the molecular mechanism of Cr(VI) carcinogenesis is poorly understood. The physico-chemical mechanism is well established and shows Cr(VI) particles being inhaled in the lungs, impacting and persisting at bronchial bifurcation sites ^64^. There the particles dissolve to release the chromate oxyanion, which is rapidly transported into cells by anion transport. Once inside the cell, Cr(VI) ions are reduced to less-stable oxidation states such as Cr(V) to Cr(IV) and ultimately to Cr(III), which accumulates inside the cell ^65, 66^. The ultimate carcinogen is unknown, but it is clear that Cr(VI) is the proximate carcinogen.

The key DNA lesions in Cr(VI) carcinogenesis are DNA double-strand breaks, which cause chromosomal instability by uncertain mechanisms. These Cr(VI)-induced breaks are primarily repaired by homologous recombination (HR) repair ^67– 71^ which maintains genomic fidelity. However, longer particulate Cr(VI) exposure inhibits the HR repair mechanism in human cells ^70, 71^. The essential HR repair effector protein RAD51 localizes in the nucleus to direct repair of particulate Cr(VI)-induced DNA double-strand breaks after acute exposure, but longer Cr(VI) exposures cause RAD51 to aberrantly accumulate in the cytoplasm, where it cannot carry out repair ^70, 71^.

How Cr(VI) exposure impacts RAD51 to inhibit HR repair is unknown, but whale cells may reveal some important insights that could lead to new understandings about human cancer. Specifically, data from North Atlantic right whale lung fibroblasts show longer exposures to Cr(VI) do not inhibit this repair despite inducing DNA damage ^72^. This outcome suggests the possibility of more effective repair in whales for this repair mechanism. Whales do have a duplication of the proliferation cell nuclear antigen ( *PCNA*) gene essential for re-synthesis of DNA during HR repair ^73^; however, the *RAD51* effect is upstream of *PCNA*, so the specific mechanism remains uncertain. Further studies elucidating these differences between human and whale HR repair could be pivotal in deepening our knowledge of Cr(VI)-induced neoplastic transformation and cancer and may provide clues about how to counter them.

### Whales inform about ecosystem health

Whales are key species in the ocean ecosystem and have a global distribution. Whales act as sentinel organisms to survey the possible effects and bioaccumulation of pollutants, including metal carcinogens (for example, Cr). For example, the research vessel *Odyssey* provided a global baseline for metal levels based on biopsies collected from the skin of free-ranging sperm whales. This pioneering work found metals are ubiquitous in all 16 regions surveyed across the ocean ^50– 54^. The data show variability in levels by region and metal.

These studies also confirmed the Cr concern indicated by earlier North Atlantic right whale findings, which reported high Cr levels in right whale biopsies ^74^. The median level of Cr in the sperm whale skin biopsies was 20.4 ppm and the highest level measured was 123 ppm, with most regions showing a mean Cr level much higher than previously reported in marine mammal tissue levels (typically less than 1 ppm) ^53^. Comparisons with humans also indicated levels were as high as the global mean in the sperm whales and were 28 times higher than the mean level observed in humans with no known Cr exposure and instead were similar to levels in lung tissue from chromate workers with lung cancer ^53^. The highest average Cr concentrations present in samples were collected near the islands of the Bahamas (80 ppm), Kiribati (40 ppm), and the Seychelles (20 ppm), whereas the lowest average Cr concentrations were observed in biopsies collected near the Canary Islands (3.7 ppm) and the coast of Sri Lanka (3.3 ppm) ^51, 53^. Biopsies collected in 2011 from adult, free-ranging female southern right whales ( *Eubalaena australis*) in the San José Gulf in Argentina showed low bioaccumulation of metals, including Cr, further showing regional variability for the ocean ecosystem ^75^.

A subsequent voyage by the research vessel *Odyssey* also tested the levels of Cr in the aftermath of the Deepwater Horizon oil rig explosion on 20 April 2010 that released over 779 million liters of crude oil into the Gulf of Mexico over the course of 87 days ^50, 51^. The expedition collected samples of resident Bryde’s whales ( *Balaenoptera edeni*) and resident sperm whales. These data showed oil-related metals (including Cr) were elevated in 2010 and declined over a three-year period after the spill ^50, 51^, implicating the marine oil industry as one potential anthropogenic source of metal pollution in the marine ecosystem.

One cannot distinguish whether exposures of these animals were to Cr(VI) or Cr(III), the other biologically stable form of Cr, because of the immediate biochemical reduction of Cr(VI) to Cr(III) inside cells. However, to reach these elevated Cr tissue levels, much of the exposure of the animals, and consequently the ecosystem surrounding them, would have had to have been to Cr(VI) because Cr(III) is poorly absorbed by all physiological routes of exposure ^58^. Consistent with such a possibility, Cr in air has been shown to contain up to 35% Cr(VI), and the chemistry of Cr in salt water favors Cr(VI) over Cr(III) ^58, 76^. Thus, given the high Cr levels and the known reproductive effects and genotoxicity of Cr(VI), the whale data indicate a significant health concern for the ocean ecosystem, as many species could be affected.

## Summary

One Environmental Health is the subset of One Health that focuses specifically on toxic chemicals. Alligators have been pivotal in understanding the field of endocrine disruption and persistent organic pollutants. Whales have been instrumental in the continued elucidation of the mechanism of carcinogenic metals and serve as sentinel organisms to measure the health of, arguably, what is our planet’s greatest resource: the ocean. These are clear examples of how one component in the One Environmental Health triad can provide key insights into the other two components of the triad and science overall.

## Abbreviations

Cr, chromium; Cr(III), trivalent chromium; Cr(VI), hexavalent chromium; DAX1, dosage-sensitive sex-reversal, adrenal hypoplasia congenita critical region on the X-chromosome 1; EDC, endocrine-disrupting chemical; HR, homologous recombination; *PCNA* , proliferation cell nuclear antigen.
